# The Local Treatment of Diseases of the Respiratory Organs

**Published:** 1887-08

**Authors:** 


### *THE LOCAL TREATMENT OF DISEASES OF THE RESPIRATORY ORGANS.*

The methods of treatment which had been employed were the direct introduction of coarse sprays, the use of the Evans inhaler and the use of the pneumatic cabinet. In the treatment of phthisis the best results are attained in the early stages of the disease, or where the disease, although further advanced, is limited to a small portion of the lung. The use of sprays is beneficial only in so far as we desire to treat the co-existent bronchitis or cavities connected with bronchi of the second or third order. In incipient phthisis with very little bronchial catarrh, local treatment is probably of little service. These cases are best treated with compressed air. In a large proportion of the cases we may hope to render the disease latent. The expansion of the lung favors the expectoration of the contents of the smaller tubes and modifies the intra-thoracic circulation. The sittings should be frequent—every day or every second day. Ten minutes is usually sufficiently long for the patient to remain in the cabinet. The pressure should gradually be increased up to one-half or three-fourths of an inch. If used cautiously this is the best method for the local treatment of incipient phthisis. With the Evans inhaler his results had also been satisfactory.

In the treatment of advanced cases the first effort must be to cure or diminish the bronchitis. The pneumatic treatment then comes into play. This gives better expansion, improves the circulation and alters the action of the trophic nerves. In all cases internal medication has been combined with the local treatment. The more acute the disease, the higher the fever and the more sudden the onset, the less can we expect to accomplish by treatment. In no case diagnosed as acute phthisis did treatment have the slightest effect.—*American Med. Journal.*

